# The predictive power of health system environments: a novel approach for explaining inequalities in access to maternal healthcare

**DOI:** 10.1136/bmjgh-2019-002139

**Published:** 2020-02-10

**Authors:** Laura Sochas

**Affiliations:** Department of Social Policy, London School of Economics and Political Science, London, UK

**Keywords:** geographic information systems, health policy, health systems, maternal health

## Abstract

**Introduction:**

The growing use of Geographic Information Systems (GIS) to link population-level data to health facility data is key for the inclusion of health system environments in analyses of health disparities. However, such approaches commonly focus on just a couple of aspects of the health system environment and only report on the average and independent effect of each dimension.

**Methods:**

Using GIS to link Demographic and Health Survey data on births (2008–13/14) to Service Availability and Readiness Assessment data on health facilities (2010) in Zambia, this paper rigorously measures the multiple dimensions of an accessible health system environment. Using multilevel Bayesian methods (multilevel analysis of individual heterogeneity and discriminatory accuracy), it investigates whether multidimensional health system environments defined with reference to both geographic and social location cut across individual-level and community-level heterogeneity to reliably predict facility delivery.

**Results:**

Random intercepts representing different health system environments have an intraclass correlation coefficient of 25%, which demonstrates high levels of discriminatory accuracy. Health system environments with four or more access barriers are particularly likely to predict lower than average access to facility delivery. Including barriers related to geographic location in the non-random part of the model results in a proportional change in variance of 74% relative to only 27% for barriers related to social discrimination.

**Conclusions:**

Health system environments defined as a combination of geographic and social location can effectively distinguish between population groups with high versus low probabilities of access. Barriers related to geographic location appear more important than social discrimination in the context of Zambian maternal healthcare access. Under a progressive universalism approach, resources should be disproportionately invested in the worst health system environments.

**Key questions**

**What is already known?**

- A range of individual-level characteristics, such as age, marital status, birth order, rural-urban residence, wealth and education are associated with facility delivery.
- The average and independent negative effect of distance and quality of care barriers on facility delivery is high.

**What are the new findings?**

- Multidimensional health system environments, incorporating both geographic and social dimensions, can accurately distinguish between population groups with high versus low probabilities of maternal healthcare access.
- Geographic dimensions of the health system environment predict access to facility delivery more accurately than dimensions linked to social discrimination.

**What do the new findings imply?**

- This study’s approach is uniquely placed to identify microenvironments where resources could be disproportionately invested under a progressive universalism approach.
- Focusing on discriminatory accuracy serves to identify specific dimensions of the health system environment that should be prioritised by policies aiming to reduce healthcare inequalities.
- Future studies of healthcare access inequalities would benefit from including comprehensive, theoretically informed models of the health system environment in their analysis.

## Introduction

Skilled, high-quality birth attendance is crucial to preventing maternal and neonatal mortality.^1^ However, inequalities in access to skilled birth attendance and facility delivery in low-income and middle-income countries (LMICs) remain larger than inequalities in other primary healthcare areas.^2^ Designing effective interventions to reduce inequalities in maternal healthcare access in LMICs is not straightforward. A review of interventions to reduce maternal and child health inequalities in LMICs found great variation: interventions can increase, decrease or fail to impact health inequalities.^3^

Better information on the determinants of maternal healthcare inequalities could help policy-makers in LMICs reduce inequalities more effectively. Many existing quantitative studies describe which types of women are less likely to access a health facility delivery according to individual characteristics such as age, wealth, education, rural-urban residence or parity, without investigating how health system environments might be shaping these disparities. These are typically data-driven analyses that rely solely on widely available household surveys (eg, Multiple Indicator Cluster Survey and Demographic Health Survey (DHS)), which measure individual characteristics but not contextual variables.^4–6^ Because such an approach erases health system characteristics as potential variables, it can implicitly ‘blame the victim’ while absolving the state from reforming health services and financing.^7 8^ This is particularly the case when authors fail to interpret individuals’ demographic characteristics as social determinants of health rooted in broader patterns of power and injustice.^9^

Merlo *et al*, in a recent article on geographic health inequalities, state that we should ‘start searching for better geographical definitions of the context that influence the (health) outcome of interest or to even combine geographical and social information to better define contexts’.^10^ The latter is precisely the context that this study attempts to capture with the concept of ‘health system environments’: the geographically and socially mediated accessibility of a local health system for the health users that surround it. The accessibility of a given health system environment should vary within a population depending on the geographic distribution of health services (facilities, staffing, levels of care) relative to the population, and depending on how inclusion and exclusion are socially patterned. For example, a given neighbourhood may be geographically close to a hospital providing high-quality care, but poor women within that neighbourhood may be discouraged from accessing care by discriminatory practices at their local facility.^11^

Linking individual-level data to health facility lists through Geographic Information Systems (GIS) enables better measurement of health system environments (eg, compared with self-reported access barrier variables in the DHS), with wide geographic reach.^12^ While the use of GIS in maternal and newborn health studies is rapidly growing,^13–15^ most studies only focus on one or two aspects of the health system environment, such as distance to care and/or quality of health services. Only by using theory to define all relevant dimensions of a health system environment and by analysing all dimensions jointly can we understand the overall relevance of the health system context in driving disparities in access, and compare the relative importance of different dimensions.

Importantly, the few studies that do consider multiple elements of the health system environment mainly use multivariable regression analysis, which reports on the average and independent effect of each covariate on facility delivery, controlling for every other covariate in the model.^12^ Multivariable regression coefficients do not take into account the distribution of facility delivery around the average for those observations where a given covariate equals one, or the overlap in the distributions for observations where the covariate equals one and for observations where the covariate equals zero.^10^ For example, while distance might be strongly and negatively associated with facility delivery, it might be that many individuals who live far away from the facility still access facility delivery (false negatives), while many of those who live close to the facility do not access (false positives). The average and independent effect of a given covariate is therefore not necessarily informative for identifying populations most in need of support.

This study aims to provide policy-relevant evidence on the structural determinants of maternal healthcare access disparities in Zambia by conducting a multilevel analysis of individual heterogeneity and discriminatory accuracy (MAIHDA). Based on currently available literature, it is the first time that (1) MAIHDA is applied outside of a high-income country context, and (2) the ‘context’ for health(care) inequalities combines the geographic and social locations of populations and health services, rather than merely neighbourhoods^10^ or intersectional social identities.^16^

Using the MAIHDA approach, this study investigates the extent to which the multidimensional health system environment within which a birth takes place is predictive of facility delivery given individual and community-level heterogeneity within those environments. It asks which dimensions of the health system environment more strongly discriminate between those who will or will not access facility delivery. In doing so, it designates groups facing health system environments that are in particular need of policy-makers’ attention if disparities are to be reduced. Each dimension of the health system environment is framed as a barrier to healthcare access in the analysis. Different combinations of these barriers define a range of potential health system environments.

This innovative approach is demonstrated using the case of Zambia. Zambia has lower levels of facility delivery (64.2% in the period 2008–14) than many countries in the Southern African region,^17^ although comparatively low levels of maternal mortality (224 deaths per 100 000 live births in 2015).^18^ Inequalities in access to facility delivery have been decreasing since 2002, yet the absolute difference between facility delivery rates for the 20% richest and 20% poorest was still almost 50 percentage points between 2008 and 2013.^17^

The Zambian Government has made it a priority to reduce these inequalities: equity of access to healthcare services was part of the mission statement and key principles of the past three National Health Strategic Plans.^19–21^ Many of the health system environment dimensions listed in the ‘Conceptual framework’ section have been documented as barriers to access in the Zambian context, in both qualitative and quantitative studies.^12 22–24^ However, quantitative studies have neither evaluated the health system environment as a whole, nor have they analysed its predictive power relative to individual and community heterogeneity. The approach demonstrated in this paper might prove particularly useful for other LMIC contexts where further progress on healthcare access inequalities is high on the agenda.

## Methods

### Conceptual framework

The dimensions of the health system environment investigated in this study are drawn from established ‘relational’ theories of healthcare access. Relational approaches conceptualise accessibility as the extent to which the health system is able to meet health users’ needs. According to these theories, the seven relevant dimensions of the health system environment are: affordability, cognitive accessibility, psychosocial accessibility, geographic accessibility, availability, perceived quality of care and administrative accessibility.^25–27^ Table 1, adapted from Choi *et al*,^28^ provides definitions for these dimensions in the left-most column and demonstrates how they relate to three existing relational theories of healthcare accessibility. Actual quality of care (as opposed to users’ perception of quality), is not part of the conceptual framework since this study is purely concerned with accessibility rather than health outcomes.

**Table 1:** Dimensions of the health system environment

Dimensions	Penchansky and Thomas^25^	Bertrand *et al* 26	UN right to health^27^
**Affordability:** ‘The relationship of prices of services to the clients’ income, ability to pay and health insurance’^25^		Economic accessibility	Accessibility (economic)
**Cognitive accessibility:** ‘Extent to which potential clients are aware of the locations of service (…) points and of the services available at these locations’^26^ Also includes the extent to which health education has been successful in explaining the benefits of quality biomedical care			Accessibility (informational)
**Psychosocial accessibility:** ‘Extent to which clients are constrained by psychological, attitudinal or social factors in seeking out (…) services’^26^ Eg, disrespect or discrimination from health workers and other patients; unacceptable care in the context of beliefs	Acceptability (attitudes of users towards providers’ personal characteristics)		Acceptability (culturally appropriate care, respecting confidentiality)
**Geographic accessibility:** ‘The relationship between the location of supply and the location of clients, taking into account client transportation resources and travel time, distance and cost’^25^			Accessibility (geographic)
**Availability:** ‘The relationship of the volume and type of existing services to the clients’ volume and types of needs’^25^			
**Perceived quality of care:** clients’ perception of the extent to which they are likely to receive effective care once they access a facility	Acceptability (user attitudes towards providers’ professional characteristics)	Quality of care	Quality of care
**Administrative accessibility:** ‘The relationship between the manner in which the supply resources are organised to accept clients and the clients’ ability to accommodate to these factors, and the clients’ perception of their appropriateness’^25^	Accommodation		

Yellow cells indicate that a theoretical framework includes that particular dimension. The text within the cells is the name given to that dimension by that theoretical framework if it differs from the name in the left-most column. Definitions are referenced where appropriate. Non-referenced definitions were developed by the author.

### Data sources

**Figure 1:**
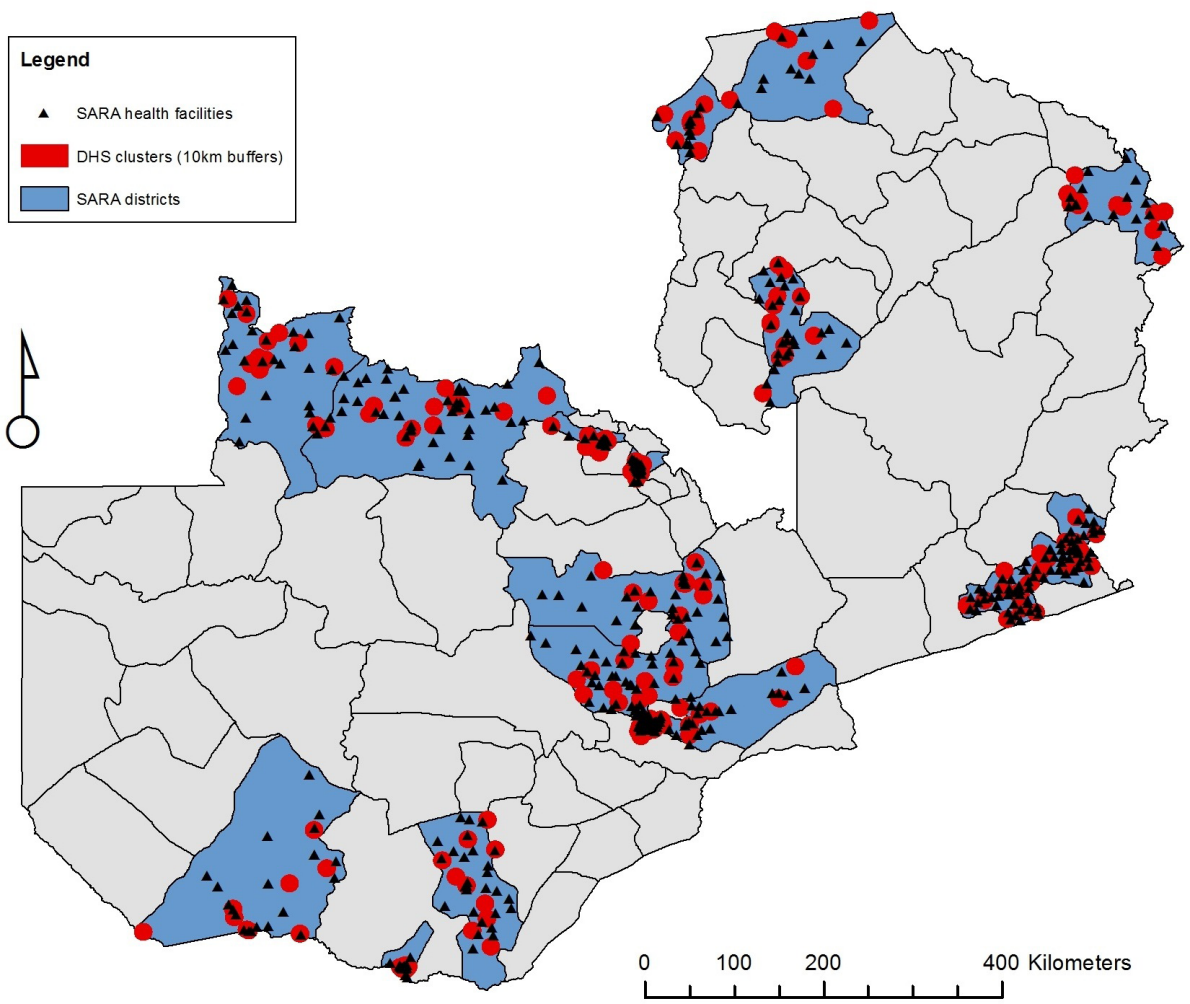
Health facilities and Demographic Health Survey (DHS) clusters in districts surveyed by the Service Availability and Readiness Assessment (SARA), Zambia. Produced by the author using ArcGIS 10.

This study uses a combination of innovative approaches, including: GIS methods to link a population-level dataset to a facility-level dataset (figure 1) and key informant interviews (KIIs) to select variables for analysis. The two main datasets are: the nationally representative 2013–14 DHS and the 2010 Service Availability and Readiness Assessment (SARA), which collected information on all facilities located in 17 of Zambia’s districts (out of 72).

The 2013–14 DHS is a cross-sectional population survey on reproductive, maternal and child healthcare access and outcomes, representative at the national and provincial levels. Individual data are de-identified and geo-referenced according to the central location of the sampling cluster, an enumeration area with an average size of 130 households. The DHS randomly displaces the geo-location of these clusters for confidentiality purposes, by 0–2 km for urban clusters and 0–5 km for rural clusters (of which 1% up to 10 km).^17^ The study sample is at the birth-level. It includes live births where the child’s mother resided within one of the 17 SARA districts, that occurred in the 5 years prior to interview (ie, those for whom place of birth information was requested during the interview), and where the sampling cluster had a valid geo-reference. Births to mothers who migrated since the birth were excluded as their residence at the time of the birth could not be obtained. Non-singleton births were excluded since they constitute a medical complication that is often identified prior to the birth, resulting in non-comparable decision-making around access to care. Observations with any missing covariates were deleted. The final sample comprises 253 clusters and 3470 live births (further details on the number of observations eliminated at each stage are provided in the online supplementary file).

10.1136/bmjgh-2019-002139.supp1 Supplementary data

The 2010 SARA collected information on health facilities’ staffing levels, drugs and equipment, from all facilities in 17 out of Zambia’s 72 districts, and geo-referenced the health facility’s location. Districts were selected evenly, but not randomly, from across Zambia’s nine provinces, in order to purposefully include malaria sentinel districts and Global Fund evaluation districts, and to include an even mix of predominantly rural and predominantly urban districts. Because of the non-random selection of districts and the fact that the DHS is not designed to be representative at the district level, this study’s sample is not statistically representative at the national level. Facilities which were revealed to be located outside the SARA districts’ shapefiles by GIS analysis,^29^ or without a valid geo-reference, were excluded. A total of 596 health facilities are included in the analysis. SARA was preferred to the Zambia 2012 health facility list, which covers all health facilities in the country, as the latter lacked sufficient information on quality of care and staffing.

Variable selection was informed by 12 KIIs, held in Lusaka in July–August 2017 with respondents from academic, government, international aid and medical backgrounds, selected purposively for their knowledge of healthcare access in Zambia. KIIs focused on the validation of the overall theoretical framework, the selection of the variables from a shortlist provided by the author, additional variable suggestions and discussion of the strengths and weaknesses of potential variables. The respondents were asked to assess potential variables according to their conceptual closeness to a given dimension and to the availability of high-quality secondary data measuring that variable in the Zambian context.

### Variables

While each of dimension of the health system environment is a complex concept, I selected one variable per dimension to avoid an exponential number of combinations and therefore health system environments, which would have caused the estimate of the probability of facility delivery for each type of health system environment to be imprecise. In order to maximise legitimacy, contextual relevance and accuracy of measurement, variable selection was informed by the KIIs described above and a Zambia-focused literature review. One dimension, administrative accessibility, could not be measured in this study, due to the lack of a suitable data source. The variables operationalising each dimension of the health system environment are binary and are conceptualised as access barriers, that is, coded as 1 if the health system environment is not conducive to healthcare access. Descriptive statistics for each variable are provided in table 2.

**Table 2:** Descriptive statistics, Zambia DHS (2013–14) and SARA (2010)

	Study sample unweighted	Original dataset weighted
	**% of births**	**% of births (DHS)**
Facility delivery	73.9	67.6
Affordability barrier *Two poorest wealth quintiles*	47.7	47.8
Cognitive barrier *Birth order 1+*	81.5	74.7
Psychosocial barrier *Birth order 6+*	25.3	16.3
	**% of births**	**% of facilities (SARA)**
Geographic barrier *No health facility within 5 km*	33.9	
*No health facility within 10 km*	21.3	
Availability barrier *No midwife*		55.9
*No midwife within 5 km*	48.9	
*No midwife within 10 km*	38.6	
Quality of care barrier *Not CEMONC*		95.1
*No CEMONC within 5 km*	72.4	
*No CEMONC within 10 km*	57.9	

CEMONC, Comprehensive Emergency Obstetric and Neonatal Care; DHS, Demographic Health Survey; SARA, Service Availability and Readiness Assessment.

*Whether a birth occurred in a health facility*, or ‘facility delivery’ for short, is the outcome variable for all analyses, and is sourced from DHS data. This variable measures whether the birth occurred at any health facility, including private and public facilities, from health posts to hospitals. Facility delivery is very closely related to being assisted by a skilled provider at birth: 95% of births in a health facility were delivered by a skilled birth attendant (SBA) (ie, doctor, clinical officer, or nurse/midwife), compared with only 0.7% of births occurring elsewhere.^17^

*The affordability barrier* is defined according to household wealth, and is coded as 1 if the mother’s household was in the two poorest wealth quintiles at the time of interview, using DHS data. Since assets that characterise wealth are different in rural versus urban contexts, wealth indices were calculated separately by the author for rural and urban residents, using principal component analysis of housing infrastructure and household assets, and then merged.^30^ This variable does not directly measure the relationship between healthcare costs and households’ financial resources, neither of which are captured by available data. However, households in the two lowest wealth quintiles are more likely to struggle to afford the cost of a facility delivery. This cost was recently estimated by a study on rural Zambia as US$29 for primary-level facilities and US$36 for hospitals, despite the absence of formal user fees, relative to an average monthly income of US$105 for the poorest rural residents.^31^ Recent qualitative research shows that facility-level expectations that mothers will bring materials for the delivery constitute a social exclusion mechanism for women without sufficient access to financial resources.^11^

*Cognitive and psychosocial barriers* are defined according to birth order, using DHS data. Birth orders above 1 are coded as facing a cognitive barrier. KIIs confirmed conclusions from the Zambian literature that multiparous mothers are less likely to view facility delivery as necessary because of their previous childbirth experience, even though complications can arise regardless of parity.^32^ Birth orders of 6 and above are coded as facing a psychosocial barrier in addition to the cognitive barrier. Key informants reported that women with six or more births are more likely to receive disrespectful care from nurses or midwives, which was confirmed in interviews conducted with mothers in Mansa district in 2018.^11^ These variables only proxy for one of the many reasons why women might face cognitive or psychosocial barriers. The extent to which high birth orders result in discrimination may vary across health facilities and health workers, but such microdata are not available.

*The geographic barrier* is defined as whether the mother’s DHS sampling cluster at the time of interview was further than 10 km from any health facility in the SARA census, measured as a straight-line distance. The last three National Health Strategic Plans (going back to 2006), all make explicit reference to the importance of increasing the percentage of the population living within 5 km of a health facility. However, because of the random displacement of DHS sampling clusters, I follow best practice and use a distance of 10 km for all geographically defined barriers in order to minimise the possibility of misclassification.^33 34^ I use straight-line distance rather than networked distance due to the noise introduced by other factors such as cluster displacement and the lack of data on means of transport to reach the health facility. I control for the cluster’s slope to partially account for the terrain and include year-month fixed effects to account for seasonality of travel time.^14 35^ By construction, any health system environment that lacks geographic accessibility also lacks the availability and perceived quality of care dimensions. This ‘nesting’ of barriers represents the reality of how the geographic, availability and quality barriers operate: one cannot have access to a skilled birth attendant or Comprehensive Emergency Obstetric Care without geographic access to a health facility (in the context of Zambia).

*The availability barrier* is defined as whether the mother’s DHS sampling cluster was further than 10 km from any health facility with a midwife, with staffing measured using SARA data. Key informants said that having a sufficient number of skilled staff was important to meet the population’s need for skilled childbirth care, which has also been emphasised in the global literature.^36^ Because SARA did not record the number of staff working in maternity care specifically, and higher-level facilities include many doctors and nurses that do not provide maternity care, I operationalised this variable to focus on midwives specifically. However, in facilities without a midwife, nurses often conduct deliveries. These facilities are still coded as having low availability, since it is assumed that a nurse is more likely to have competing demands on her time beyond delivery care, and availability pertains to the balance between the volume of need and services provided. By construction, any health system environment that lacks availability also lacks the perceived quality of care dimension.

*The perceived quality of care barrier* is defined as whether the mother’s DHS sampling cluster at the time of interview was further than 10 km from any health facility with the capacity to provide Comprehensive Emergency Obstetric and Neonatal Care (CEMONC). A CEMONC facility is able to respond to all obstetric complications, including those requiring caesarean section and blood transfusion, and is thus able to save lives when complications arise in childbirth.^37^ CEMONCs were identified in the SARA data according to whether the facility’s manager reported that the facility provided all eight CEMONC signal functions. Reporting was based on the question: ‘Which of the following obstetric care services does this facility provide?’, combined with a list of signal functions, for example, ‘parenteral administration of antibiotics’ and Yes/No answers for each type of service.^38^ Among the facilities included in this study, all facilities coded as providing CEMONC are hospitals, although only 76% of hospitals provided CEMONC. While this variable is likely to overestimate facilities’ practical ability to carry out signal functions, and while quality of care goes far beyond signal functions, a CEMONC facility is more likely to be *perceived* by lay persons to provide quality care.^39 40^

### Analytical strategy

This study applies an innovative method from social epidemiology—MAIHDA.^41 42^ This approach has two key advantages. First, it takes into account the mean average effect of different dimensions of the health system environment on the outcome, and the distribution of the outcome within and between groups facing different types of health system environments. This allows the study to estimate the predictive power of the health system environment relative to individual and community heterogeneity.^42–44^ Second, the MAIHDA approach allows for a more precise estimation of the predicted probability of facility delivery for births in each health system environment, since probabilities for rare combinations are estimated by borrowing information from the mean.^41^ Since this method has been extensively described in other authors’ publications, further technical details are provided in the online supplementary file.

In this study, MAIHDA is implemented using a binomial logistic random intercepts model. Births are nested within one of 24 mutually exclusive health system environments, defined according to all feasible combinations of the relevant dimensions or barriers (table 3). The number of combinations allows for the fact that some barriers cannot be experienced without others. A random intercept is specified for each of the 24 health system environments. In the baseline model, the barrier variables are only represented using random intercepts and are not included as explanatory variables: the non-random part of the model remains empty, apart from control variables where relevant. The intraclass correlation coefficient (ICC) calculates the percentage of the total variance attributable to the health system environment, relative to individual-level variance (and community-level variance, where relevant). The higher the ICC, the more accurately the health system environment as a whole can predict who will and who will not access a facility delivery.

**Table 3:** Predicted probability of facility delivery for women facing different health system environments, Zambia 2013–14

#	Births N	Births* %	Barriers N	Affor	Cogn	Psyc	Geog	Avail	Qual	Pred prob	CI
1	214	6	6	Yes	Yes	Yes	Yes	Yes	Yes	0.41	0.34 to 0.48
2	271	8	5	Yes	Yes	No	Yes	Yes	Yes	0.42	0.35 to 0.48
3	90	3	4	No	Yes	No	Yes	Yes	Yes	0.49	0.39 to 0.60
4	67	2	5	No	Yes	Yes	Yes	Yes	Yes	0.52	0.40 to 0.64
5	160	5	5	Yes	Yes	Yes	No	Yes	Yes	0.52	0.44 to 0.60
6	230	7	4	Yes	Yes	No	No	Yes	Yes	0.60	0.53 to 0.66
7	75	2	4	Yes	No	No	Yes	Yes	Yes	0.60	0.49 to 0.71
8	47	1	4	No	Yes	Yes	No	Yes	Yes	0.64	0.49 to 0.78
9	105	3	4	Yes	Yes	Yes	No	No	Yes	0.66	0.56 to 0.75
10	59	2	3	Yes	Yes	Yes	No	No	No	0.66	0.54 to 0.78
11	22	1	3	No	No	No	Yes	Yes	Yes	0.67	0.48 to 0.84
12	71	2	3	No	Yes	Yes	No	No	Yes	0.72	0.61 to 0.83
13	225	6	3	Yes	Yes	No	No	No	Yes	0.72	0.66 to 0.79
14	64	2	3	Yes	No	No	No	Yes	Yes	0.78	0.68 to 0.88
15	62	2	2	Yes	No	No	No	No	Yes	0.82	0.72 to 0.91
16	154	4	2	Yes	Yes	No	No	No	No	0.82	0.76 to 0.88
17	153	4	2	No	Yes	No	No	No	Yes	0.83	0.77 to 0.89
18	29	1	2	No	No	No	No	Yes	Yes	0.84	0.72 to 0.95
19	71	2	3	No	Yes	No	No	Yes	Yes	0.84	0.75 to 0.93
20	155	4	2	No	Yes	Yes	No	No	No	0.86	0.80 to 0.91
21	37	1	1	Yes	No	No	No	No	No	0.90	0.80 to 0.98
22	758	22	1	No	Yes	No	No	No	No	0.93	0.91 to 0.95
23	299	9	0	No	No	No	No	No	No	0.94	0.92 to 0.97
24	55	2	1	No	No	No	No	No	Yes	0.96	0.91 to 1.00

*% of births is unweighted.

Affor, affordability barrier; Avail, availability barrier; CI, 95% Bayesian credible intervals; Cogn, cognitive barrier; Geog, geographic barrier; Psych, psychosocial barrier; Qual, quality barrier.

I then explore which dimensions of the health system environment have stronger discriminatory accuracy by comparing the ICC of the environments’ random intercepts in an otherwise empty model (described above) versus a range of models that also include the barrier variables in the non-random part of the model.^16^ Once the variable for a given barrier is included in the non-random part of the model, the variance of the environments’ random intercepts no longer captures the additive effect of that barrier variable, and is reduced. The larger the proportional difference between the random intercepts’ variance in the two models, the more discriminatory accuracy that dimension or barrier has. I estimate all models using Bayesian Markov Chain Monte Carlo methods, as recommended in the MAIHDA literature (see online supplementary file for details).^41^ Bayesian statistics do not produce frequentist measures of statistical significance, such as t-statistics and P-values. Uncertainty is communicated using 95% Bayesian credible intervals: there is a 95% probability that the parameter of interest is contained with the credible interval.

I include an additional, cross-classified random intercept at the DHS sampling cluster level in sensitivity analyses. This allows for a better estimate of the uncertainty of point estimates, by accounting for the fact that births within mothers and mothers within clusters are likely to be more similar to each other than to births from different mothers or in different clusters. This random effect also represents community-level heterogeneity, which is of substantive interest. In order for the model to accurately partition the variance between the two cross-classified random effects, there must be a sufficient degree of interpenetration between membership of the community (cluster) and membership of the health system environment. While the geographic, availability and quality of care dimensions do not vary by cluster, the other three dimensions do, making a total of six potential health system environments within each cluster. According to Vassallo *et al*,^45^ this is a sufficient level of interpenetration between levels. Where a cluster-level random intercept is included, the calculation of the ICC includes the variance of this new random intercept in the denominator. I also include individual-level control variables shown to be associated with facility delivery^8^: marital status (a dummy for being married), educational achievement (a dummy for having reached secondary school or above) and age of the mother at birth (continuous variable in years). Other controls are related to the distance barrier: how steep the terrain of the sampling cluster is, and seasonality of time of birth (fixed effects for month-year of birth). I do not include rural-urban residence as a control variable because it is collinear with the quality of care barrier.

### Limitations

This analysis presents a number of limitations. Some of the variables chosen to measure each dimension measure only one part of that concept, leaving other parts unaddressed. This is particularly true for the cognitive and psychosocial dimensions. This limitation is the corollary of building a parsimonious model with a sufficient number of combinations to allow the variance of the environments’ random effects to be reliably estimated, while allowing for few enough environments to predict probabilities for each environment accurately. This limitation was partly addressed by drawing on a literature review and primary qualitative research to operationalise variables for the Zambian context, in order to maximise the legitimacy and contextual relevance of the variables chosen.

The variance of the random effects may be capturing the influence of omitted variables correlated both with the environment and the outcome variable. Control variables and cluster-level random effects were included in the model in order to partially address this bias. The theoretical grounding of the model also addresses this limitation, by guiding the inclusion of all major dimensions of accessibility in a single model. Only one major dimension could not be included due to lack of data: administrative accessibility.

DHS clusters are randomly displaced to maintain participant confidentiality. Some births will have been mistakenly classified as suffering from the geographic, availability or quality barriers when they did not, and vice versa. The direction of this bias cannot be predicted. In order to partially address this issue, I define distance-related variables at the 10 km level.^33 34^

### Patientand public involvement

There were no funds or time allocated for patient and public involvement in this doctoral study, such that I was unable to involve the public. However, the views of women who had recently given birth in the Zambian health system were collected, analysed and separately published as part of the same research project.

## Results

In this section, I investigate whether the health system environment is predictive of facility delivery. Conditional on this result, I explore which health system environments predict particularly low access. Finally, I examine whether there are aspects of the health system environment that are more predictive than others, and which dimensions are particularly important.

### Discriminatory accuracy of the health system environment

In the most robust model, which operationalises barriers using 10 km variables, controls for confounders and accounts for community heterogeneity, 25% of the total variance in facility delivery is explained by the variance between health system environments (model 3, table 4). The variance in facility delivery between births facing different health system environments is estimated at 1.56 (for which the 95% Bayesian credible intervals do not include zero). This is larger than the variance in facility delivery between ‘communities’ (operationalised according to DHS sampling clusters), estimated at 1.30. The remainder of the variance is that between individuals, which is fixed at 3.29 for binomial logistic models.

**Table 4:** Intraclass correlations for health system environments, Zambia 2013–14

	No controls No cluster RE	No controls With cluster RE	With controls With cluster RE
**10 km variables**	**(1)**	**(2)**	**(3)**
ICC HS environments	27%	27%	25%
ICC components:			
Variance HS environments	1.20 (0.50 to 2.10)	1.59 (0.62 to 2.82)	1.56 (0.56 to 2.83)
Variance communities	NA	1.10 (0.72 to 1.51)	1.30 (0.85 to 1.78)
Variance individuals	3.29	3.29	3.29
**5 km variables**	**(4)**	**(5)**	**(6)**
ICC HS environments	26%	25%	22%
ICC components:			
Variance HS environments	1.13 (0.48 to 1.96)	1.50 (0.58 to 2.65)	1.36 (0.48 to 2.46)
Variance communities	NA	1.22 (0.83 to 1.66)	1.43 (0.95 to 1.93)
Variance individuals	3.29	3.29	3.29

The ICC indicates the proportion of the variance in facility delivery that can be explained by the variance between HS environments, controlling for confounders and accounting for clustering within DHS sample clusters. Individual-level variance is set at 3.29 for binomial logistic models (95% Bayesian credible intervals in parentheses).

Controls: mothers’ age at birth, married, secondary school or higher, cluster slope, month-year fixed effects.

Cluster RE model also includes a cross-classified random intercept for DHS sampling clusters in addition to the environments’ random intercepts.

5 km variables: geographic, availability and quality variables defined at the 5 km level—others defined as normal.

10 km variables: geographic, availability and quality variables defined at the 10 km level—others defined as normal.

DHS, Demographic Health Survey; HS, health system; ICC, intraclass correlation coefficient; NA, not available; RE, random effects.

An ICC of 25% represents a high level of discriminatory accuracy, or predictive power: Axelsson Fisk *et al*,^16^ drawing on cut-offs used in psychometric test reliability assessments, suggest that an ICC of 20%–30% is ‘very good’, while Merlo *et al* 10 state that 20–30 points to ‘fairly large’ differences between groups.

### Which health system environments predict low facility delivery?

Results show that 91% of the sample face health system environments with at least one barrier, while 6% of the sample live in a health system environment where all six barriers are present (table 3, unweighted). There are wide disparities in the probability of accessing a facility delivery depending on the health system environment. Unsurprisingly, women living in a health system environment with all six barriers have the lowest chance of giving birth in a health facility (41% probability), while women facing an environment with no barriers have a 94% probability of doing so. All births facing four barriers or more (combinations #1–#9; 37% of the sample) have a predicted probability of facility delivery that is below average (73.9% in the study sample, unweighted) (table 3).

With some exceptions, health system environments with fewer barriers have a higher predicted probability of facility delivery than environments with a greater number of barriers. Exceptions are likely explained by the uncertainty of the point estimates, described by the credible intervals in the right-most column, as well as the particularly strong contributions of some barriers (eg, geographic accessibility). In general, there are larger disparities between health system environments where the number of barriers is different, compared with disparities between health system environments with the same number of barriers but where the specific barriers faced are different.

### Do some aspects of the health system environment matter more?

The analysis presented above allows policy-makers to accurately identify population groups that are particularly at risk of not giving birth in a health facility. As a next step, investigating whether specific dimensions of the health system environment are particularly predictive of facility delivery could help policy-makers prioritise these dimensions for improvement.

The inclusion of the affordability, cognitive and psychosocial dimensions in the non-random part of the model (in separate models) reduces the variance of the environments’ random effects by 15% or less (models 2–4, table 5), compared with 47% or more for the geographic, availability and quality barriers (models 5–7, table 5). The greater predictive power of these last three dimensions is confirmed by comparing the change in the variance when the first three barriers are all included in the non-random part of the model (a change of −27%) (model 8, table 5), relative to when the last three barriers are all included (a change of −74%) (model 9, table 5).

**Table 5:** Comparing the discriminatory accuracy of different dimensions within the health system environment using the proportional change in variance, Zambia 2013–14 (binomial logistic random intercepts model)

Facility delivery	(1)	(2)	(3)	(4)	(5)	(6)	(7)	(8)	(9)
	Reference model	Afford	Cogn	Psych	Geog	Avail	Qual	Afford+ cogn+ psych	Geog+ avail+ qual
ICC	25%	23%	22%	25%	14%	13%	15%	20%	8%
PCV	Reference model	−12%	−15%	−4%	−52%	−54%	−47%	−27%	−74%
Variance: HS environments	1.6	1.4	1.3	1.5	0.7	0.7	0.8	1.1	0.4
	(0.6 to 2.8)	(0.5 to 2.6)	(0.5 to 2.5)	(0.5 to 2.8)	(0.2 to 1.4)	(0.2 to 1.4)	(0.2 to 1.6)	(0.3 to 2.2)	(0.1 to 0.9)
Variance: DHS clusters	1.3	1.3	1.3	1.3	1.3	1.3	1.4	1.3	1.3
	(0.8 to 1.8)	(0.9 to 1.8)	(0.8 to 1.8)	(0.9 to 1.8)	(0.9 to 1.8)	(0.9 to 1.8)	(0.9 to 1.9)	(0.8 to 1.8)	(0.9 to 1.8)
Additive effects *(logit coeffs)*									
Afford		−0.8						−0.9	
		(−1.9 to 0.2)						(−1.8 to 0.1)	
Cogn			−1.2					−1.1	
			(−2.3 to −0.1)					(−2.2 to 0.1)	
Psych				−0.9				−0.3	
				(−2 to 0.3)				(−1.5 to 0.8)	
Geog					−2.0				−1.2
					(−3 to −1)				(−2.2 to −0.2)
Avail						−1.7			−0.6
						(−2.6 to −0.9)			(−2.2 to −0.2)
Qual							−1.8		−1.0
							(−2.8 to −0.8)		(−1.9 to −0.1)
**Constant**	−8.7	9.2	0.5	−1.6	1.4	4.2	11.4	−0.9	4.2
	(−17.3 to 1.5)	(−6.4 to 21.8)	(−8.6 to 10.8)	(−9.9 to 9.4)	(−6.3 to 9.8)	(−8.2 to 20.1)	(−1.4 to 23.4)	(−13.8 to 7.8)	(−5.9 to 14.1)

Including a barrier variable in the non-random part of the model in addition to the random part ensures that the HS environments REs’ variance no longer accounts for the additive effect of that variable. This analysis shows the extent to which the ICC decreases with the inclusion of each dimension. A greater decrease in the ICC (and a correspondingly large PCV) indicates that a specific barrier contributes more strongly to the HS environments’ collective discriminatory accuracy. 95% Bayesian credible intervals in parentheses. Controls included in this analysis: mothers’ age at birth, married, secondary school or higher, cluster slope, month-year fixed effects. The model also includes a cross-classified random intercept for DHS sampling clusters in addition to the environments’ random intercept. Individual-level variance is set at 3.29.

Afford, affordability; Avail, availability; Cogn, cognitive; DHS, Demographic Health Survey; Geog, geographic; HS, health system; ICC, intraclass correlation coefficient; PCV, Proportional Change in Variance; Psych, psychosocial; Qual, quality; RE, random effects.

## Discussion

This study uses geo-referenced population-level and facility-level datasets to rigorously measure the multiple dimensions of an accessible health system environment. It then uses random intercepts as part of an innovative approach, MAIHDA, to investigate whether multidimensional health system environments can reliably predict facility delivery.

This study shows that health system environments meaningfully predict which births are most or least likely to take place in a health facility in Zambia, even when controlling for common individual-level determinants and taking into account residual differences between individuals and communities facing similar health system environments. Given that the health system environment reliably organises the population into groups that are differentially likely to access facility delivery, policy-makers may want to know which types of health system environments are particularly discouraging. The predicted probabilities of facility delivery for each health system environment show clearly that the environments predicting lower levels of facility delivery are generally those characterised by a greater number of barriers. Environments with four or more barriers are particularly likely to be disadvantaged. Under a progressive universalism approach, these types of health system environments should be improved as a priority.^46^

The geographic, availability and quality of care dimensions are particularly predictive of access to facility delivery in Zambia. This implies that aspects of the health system environment linked to the geographic location of infrastructure, staffing and other resources required for high-quality care predicts access more strongly than exclusion linked to patients’ financial resources, their parity or unaddressed misconceptions. These dimensions also ‘hang together’ from a common-sense (and evidence-based) perspective, since it would be ill-advised to build new health facilities without staff, drugs, equipment or infrastructure.^47^ From a theoretical perspective, the geographical relationship between the health system and the population appears to more strongly structure who accesses healthcare than social location, which indicates implicit or explicit social exclusion within the health system. The results could also be affected by measurement limitations. Data constraints meant that the affordability, cognitive and psychosocial dimensions were crudely measured using individual characteristics that we know tend to be discriminated against by the existing health system, rather than data on geographic proximity to discriminating providers or facilities.

This study’s results are consistent with Gabrysch *et al*,^12^ who analyse the average and independent effect of distance and quality of care barriers (which is defined to include staffing) on facility delivery in Zambia in 2002–07, controlling for household wealth and birth order, among other confounders. The authors conclude that under a causal interpretation, ensuring that all women live within 5 km of a basic emergency obstetric care facility with appropriate staffing would reduce the proportion of home deliveries by a greater extent than if all households were in the richest wealth quintile.

The health system environments defined in this paper reflect a relational and multidimensional view of the context of health inequalities, linking health system resources, the geographic distribution of these resources relative to the population and the overt or implicit social exclusion of women inhabiting certain social locations. This frame encourages policy-makers to ask new questions in their efforts to address disparities: *Where* to build new facilities or send additional midwives, drugs and equipment? *Which* groups are still unable to afford a facility delivery even after the abolition of user fees? *Which* groups’ misconceptions remain unaddressed by health education? *Which* groups experience discrimination within the health system? By linking geographic and social locations, health system and patient characteristics, this study also demonstrates the contribution that social epidemiology can bring to health policy. The framework adopted in this paper is strongly influenced by ecosocial theory, which links multiple levels of analysis to enhance our understanding of health inequities,^48^ while the MAIHDA methodology has been developed within the field of (intersectional) social epidemiology.^42^

Gathering additional data on the cognitive and psychosocial dimensions would improve the reliability of future analyses. In the Zambian context, this could involve gathering data on how maternal health information is understood and interpreted by women and their families and on stigmatising staff attitudes. Further research with important implications for equity could build on this study to explore the extent to which inequalities defined by a range of demographic characteristics (eg, high vs low education; rural vs urban residence) are explained by the different dimensions of the health system environment, using decomposition methods.^49^ While this study focuses on healthcare access, the approach used in this paper could be extended to study inequalities in health(care) outcomes or well-being. In contrast with healthcare access, social location might prove more important in driving these other types of inequalities, because of the social nature of healthcare interactions.^50^

## Conclusion

Health system environments, defined according to the geographic and social locations of health system resources and the populations they serve, can meaningfully predict which births will take place in health facilities and which ones will not. This approach generates important information for policy-makers or activists seeking to reduce disparities in maternal healthcare access. Findings identified the worst health system environments, where resources could be disproportionately invested under a progressive universalism approach. Specific dimensions of the health system environment, that is, geographic accessibility, availability and perceived quality of care, were identified as having particularly strong discriminatory accuracy and should be considered a priority for policies aiming to reduce maternal healthcare inequalities in Zambia.

## References

[1] Miller S, Abalos E, Chamillard M, et al Beyond too little, too late and too much, too soon: a pathway towards evidence-based, respectful maternity care worldwide. The Lancet 2016;388:2176–92. 10.1016/S0140-6736(16)31472-6 27642019

[2] Boerma T, Requejo J, Victora CG, et al Countdown to 2030: tracking progress towards universal coverage for reproductive, maternal, newborn, and child health. The Lancet 2018;391:1538–48. 10.1016/S0140-6736(18)30104-1 29395268

[3] Yuan B, Målqvist M, Trygg N, et al What interventions are effective on reducing inequalities in maternal and child health in low- and middle-income settings? A systematic review. BMC Public Health 2014;14:634 10.1186/1471-2458-14-634 24952656 PMC4083351

[4] Asrese K, Adamek ME Women’s social networks and use of facility delivery services for uncomplicated births in North West Ethiopia: a community-based case-control study. BMC Pregnancy Childbirth 2017;17:441 10.1186/s12884-017-1626-8 29282081 PMC5745854

[5] Nghargbu R, Olaniyan O Inequity in maternal and child health care utilization in Nigeria. Afr Dev Rev 2017;29:630–47. 10.1111/1467-8268.12301

[6] Amo-Adjei J, Aduo-Adjei K, Opoku-Nyamah C, et al Analysis of socioeconomic differences in the quality of antenatal services in low and middle-income countries (LMICs). PLoS One 2018;13:1–12. 10.1371/journal.pone.0192513 PMC5825027 29474362

[7] Desai S Maternal education and child health: a feminist dilemma. Feminist Studies 2000;26:425–46. 10.2307/3178543

[8] Gabrysch S, Campbell OMR Still too far to walk: literature review of the determinants of delivery service use. BMC Pregnancy Childbirth 2009;9 10.1186/1471-2393-9-34 PMC2744662 19671156

[9] Marmot M, Friel S, Bell R, et al Closing the gap in a generation: health equity through action on the social determinants of health. The Lancet 2008;372:1661–9. 10.1016/S0140-6736(08)61690-6 18994664

[10] Merlo J, Wagner P, Leckie G A simple multilevel approach for analysing geographical inequalities in public health reports: the case of municipality differences in obesity. Health Place 2019;58:102145 10.1016/j.healthplace.2019.102145 31195211

[11] Sochas L Women who break the rules: social exclusion and inequities in pregnancy and childbirth experiences in Zambia. Soc Sci Med 2019;232:278–88. 10.1016/j.socscimed.2019.05.013 31112919

[12] Gabrysch S, Cousens S, Cox J, et al The influence of distance and level of care on delivery place in rural Zambia: a study of linked national data in a geographic information system. PLoS Med 2011;8:e1000394 10.1371/journal.pmed.1000394 21283606 PMC3026699

[13] Ebener S, Guerra-Arias M, Campbell J, et al The geography of maternal and newborn health: the state of the art. Int J Health Geogr 2015;14 10.1186/s12942-015-0012-x PMC4453214 26014352

[14] Makanga PT, Schuurman N, von Dadelszen P, et al A scoping review of geographic information systems in maternal health. Int J Gynaecol Obstet 2016;134:13–17. 10.1016/j.ijgo.2015.11.022 27126906 PMC4996913

[15] Matthews Z, Rawlins B, Duong J, et al Geospatial analysis for reproductive, maternal, newborn, child and adolescent health: gaps and opportunities. BMJ Glob Health 2019;4:e001702 10.1136/bmjgh-2019-001702 PMC6606071 31321094

[16] Axelsson Fisk S, Mulinari S, Wemrell M, et al Chronic obstructive pulmonary disease in Sweden: an intersectional multilevel analysis of individual heterogeneity and discriminatory accuracy. SSM Popul Health 2018;4:334–46. 10.1016/j.ssmph.2018.03.005 29854918 PMC5976844

[17] Central Statistical Office (CSO) [Zambia], Ministry of Health (MOH) [Zambia], ICF International Zambia demographic and health survey 2013-14. Rockville, Maryland, USA, 2014 Available: https://dhsprogram.com/publications/publication-FR304-DHS-Final-Reports.cfm

[18] WHO, UNICEF, UNFPA, *et al* Trends in maternal mortality: 2000 to 2017, 2019 Available: https://www.who.int/reproductivehealth/publications/maternal-mortality-2000-2017/en/

[19] Republic of Zambia Ministry of Health National health strategic plan 2006–2010, 2009

[20] Republic of Zambia Ministry of Health National health strategic plan 2017–2021, 2017 Available: http://www.moh.gov.zm/docs/ZambiaNHSP.pdf

[21] Republic of Zambia Ministry of Health National health strategic plan 2011–2015, 2011 Available: http://www.moh.gov.zm/docs/nhsp.pdf

[22] Mutale W, Bond V, Mwanamwenge MT, et al Systems thinking in practice: the current status of the six WHO building blocks for health system strengthening in three BHOMA intervention districts of Zambia: a baseline qualitative study. BMC Health Serv Res 2013;13:291 10.1186/1472-6963-13-291 23902601 PMC3734193

[23] Sialubanje C, Massar K, Hamer DH, et al Understanding the psychosocial and environmental factors and barriers affecting utilization of maternal healthcare services in Kalomo, Zambia: a qualitative study. Health Educ Res 2014;29:521–32. 10.1093/her/cyu011 24663431

[24] Sialubanje C, Massar K, Hamer DH, et al Personal and environmental predictors of the intention to use maternal healthcare services in Kalomo, Zambia. Health Educ Res 2014;29:1028–40. 10.1093/her/cyu057 25274723

[25] Penchansky R, Thomas JW The concept of access: definition and relationship to consumer satisfaction. Med Care 1981;19. 10.1097/00005650-198102000-00001 7206846

[26] Bertrand JT, Hardee K, Magnani RJ, et al Access, quality of care and medical barriers in family planning programs. Int Fam Plan Perspect 1995;21:64–74. 10.2307/2133525

[27] UN CESCR General Comment No.14: the right to the highest attainable standard of health (Art.12) 2000;2000.

[28] Choi Y, Fabic MS, Adetunji J Measurement of Access to Family Planning in Demographic and Health Surveys : Lessons and Challenges. In: PFRH Seminar, JHSPH. USAID 2014.

[29] Hijmans R Zambia ESRI file geodatabase. Glob. Adm. Areas, v.2.8 2015 www.gadm.org (accessed 13 Jun 2016).

[30] Filmer D, Pritchett L Estimating Wealth Effects without Expenditure Data-or Tears : An Application to Educational Enrollments in States of India. Demography 2001;38:115–32. 11227840 10.1353/dem.2001.0003

[31] Kaiser JL, McGlasson KL, Rockers PC, et al Out-Of-Pocket expenditure for home and facility-based delivery among rural women in Zambia: a mixed-methods, cross-sectional study. Int J Womens Health 2019;11:411–30. 10.2147/IJWH.S214081 31447591 PMC6682766

[32] Mulenga T, Moono M, Mwendafilumba M, et al Home deliveries in the capital: a qualitative exploration of barriers to institutional deliveries in peri-urban areas of Lusaka, Zambia. BMC Pregnancy Childbirth 2018;18 10.1186/s12884-018-1837-7 PMC5984831 29859063

[33] Burgert CR, Prosnitz D Linking DHS household and SPA facility surveys: data considerations and geospatial methods. DHS Spat Anal Reports;10.

[34] Wang W, Winter R, Mallick L, et al The relationship between the health service environment and service utilization: linking population data to health facilities data in Haiti and Malawi 2015.

[35] DHS Program Zambia 2013-14 demographic health survey spatial covariates. ZMGC62FL, 2017 Available: https://spatialdata.dhsprogram.com/covariates/ [Accessed 25 Feb 2019].

[36] Downe S, Matthews Z, Homer CSE, et al Improvement of maternal and newborn health through midwifery. Lancet 2014;384:1226–35. 24965818 10.1016/S0140-6736(14)60930-2

[37] Freedman LP, Graham WJ, Brazier E, et al Practical lessons from global safe motherhood initiatives: time for a new focus on implementation. The Lancet 2007;370:1383–91. 10.1016/S0140-6736(07)61581-5 17933654

[38] WHO, UNFPA, Unicef, *et al* Monitoring emergency obstetric care, a Handbook 2009.

[39] Kruk ME, Mbaruku G, McCord CW, et al Bypassing primary care facilities for childbirth: a population-based study in rural Tanzania. Health Policy Plan 2009;24:279–88. 10.1093/heapol/czp011 19304785

[40] Siam ZA, McConnell M, Golub G, et al Accuracy of patient perceptions of maternity facility quality and the choice of providers in Nairobi, Kenya: a cohort study. BMJ Open 2019;9:e029486–7. 10.1136/bmjopen-2019-029486 PMC6677992 31366657

[41] Evans CR, Williams DR, Onnela J-P, et al A multilevel approach to modeling health inequalities at the intersection of multiple social identities. Soc Sci Med 2017:0–1. 10.1016/j.socscimed.2017.11.011 29199054

[42] Merlo J Multilevel analysis of individual heterogeneity and discriminatory accuracy (MAIHDA) within an intersectional framework. Soc Sci Med 2017. 10.1016/j.socscimed.2017.12.026 29305018

[43] Austin PC, Merlo J Intermediate and advanced topics in multilevel logistic regression analysis. Stat Med 2017;36:3257–77. 10.1002/sim.7336 28543517 PMC5575471

[44] Wemrell M, Mulinari S, Merlo J An intersectional approach to multilevel analysis of individual heterogeneity (MAIH) and discriminatory accuracy. Soc Sci Med 2017;178:217–9. 10.1016/j.socscimed.2017.02.040 28274599

[45] Vassallo R, Durrant G, Smith P Separating interviewer and area effects by using a cross-classified multilevel logistic model: simulation findings and implications for survey designs. J R Stat Soc A 2017;180:531–50. 10.1111/rssa.12206

[46] Gwatkin DR, Ergo A Universal health coverage: friend or foe of health equity? The Lancet 2011;377:2160–1. 10.1016/S0140-6736(10)62058-2 21084113

[47] Campbell OMR, Calvert C, Testa A, et al The scale, scope, coverage, and capability of childbirth care. The Lancet 2016;388:2193–208. 10.1016/S0140-6736(16)31528-8 27642023

[48] Krieger N Theories for social epidemiology in the 21st century: an ecosocial perspective. Int J Epidemiol 2001;30:668–77. 10.1093/ije/30.4.668 11511581

[49] Sochas L A decomposition analysis of inequalities in maternal healthcare access in Zambia using healthcare access barriers. In: BSPS Annual Conference, 2019-09-09 - 2019-09-11, University Hall Cardiff University, 2019 http://eprints.lse.ac.uk/101032/

[50] Ramírez V CCTs through a wellbeing lens: the importance of the relationship between front-line officers and participants in the Oportunidades/Prospera programme in Mexico. Soc Policy Soc 2016;15:451–64. 10.1017/S1474746416000129

