# Supplementary material for: The predictive power of health system environments: a novel approach for explaining inequalities in access to maternal healthcare
Source: BMJ Glob Health. 2020 Feb 10;4(Suppl 5):e002139. doi: 10.1136/bmjgh-2019-002139 (PMC7044705; doi:10.1136/bmjgh-2019-002139)
Supplement: Supplementary data [file bmjgh-2019-002139supp001.pdf]

SUPPLEMENTARY FILE

Sample selection

In the DHS dataset, births to mothers who migrated since the birth were excluded, as their residence at the time of the birth could not be obtained (21,034 excluded out of an original sample of 49,207). Non-singleton births were excluded since they constitute a medical complication that is often identified prior to the birth, such that the determinants of access to care in childbirth are fundamentally different to non-singleton births (496 excluded out of 28,173). Births that occurred prior to 2008 were excluded, as the location of the birth was not recorded in the survey (16,392 excluded out of 27,677). Births that did not have a valid geo-reference were excluded (two sampling clusters and 45 births out of 11,285). Births that were not located in one of the 17 SARA districts were excluded (466 sampling clusters and 7,671 births out of 11,240). Finally, observations with missing values on covariates were excluded (99 out of 3569), leaving a final sample of 3,470 observations.

In the SARA dataset, originally composed of 658 facilities, 17 facilities were dropped due to having no or incorrect geo-references and 45 were excluded due to being identified as located outside of the SARA districts' shapefiles through GIS analysis. The final sample was made up of 596 facilities.

Multilevel Analysis of Individual Heterogeneity and Discriminatory Accuracy (MAIHDA)

MAIHDA is implemented using a logistic random intercepts model (Equation 1):

Equation 1: Baseline logistic random intercept model

$$\text{logit}\{\Pr(\mathrm{y_{ijz}} = 1|\theta_{izj}, \mathrm{\mu_{1j}}, \mathrm{\mu_{2j}})\} = \propto\ + \theta_{izj} +\ \mathrm{\mu_{1j}} + \mu_{2z}$$

Where:

$$\mathrm{\mu_{1j}} \sim N(0, \varphi_1)$$

$$\mathrm{\mu_{2z}} \sim N(0, \varphi_2)$$

Where: y_{ijz} is facility delivery for the ith birth nested in both the jth community (i.e.: Demographic and Health Survey sampling clusters) and the zth health system environment. α is the overall mean of facility delivery. θ is a vector of control variables.

The two sets of random intercepts μ_{1j} and μ_{2j} are assumed to be normally distributed with mean zero and uncorrelated with each other. The community random intercepts μ_{1j} have variance φ_1, are independent and identically distributed across communities, and are independent from control variables θ_{ijz}. The health system environment random intercepts μ_{2z} have variance φ_2, are independent and identically distributed across health system environments, and are independent from control variables θ_{ijz} .[1]

Using such a model, the predicted probability of a facility delivery can be estimated for each health system environment. These probabilities are more reliably estimated in a multi-level model than a saturated fixed-effects model, since probabilities for rare combinations are estimated by borrowing information from the mean.[2]

The health system environments' ICC is calculated as the share of the variance attributable to the health system environment random intercepts' variance , φ_2 , relative to the total variance, made up of the health system environment random intercepts' variance φ_2, the community random intercepts' variance φ_1, and the individual-level variance, which is set at 3.29 in binomial logistic models (Equation

Sochas L. *BMJ Global Health* 2020; S:e002139. doi: 10.1136/bmjgh-2019-002139

2). The ICC measures the level of discriminatory accuracy, similar to the Area Under the Receiver operating characteristic curve (AUC).[3] The higher the ICC, the better the barrier combinations are at distinguishing between who will and will not access a facility delivery.

Equation 2: Intraclass Correlation Coefficient $\text{ICC} = \frac{\varphi_2}{\varphi_1 + \varphi_2 + 3.29}$

In subsequent models, I explore which dimensions have the most discriminatory accuracy by comparing the ICC of the health system environments' random intercepts in Equation 1 (model A) versus the ICC of the same random intercepts in a model that also includes barrier variable dummies as main effects (model B). This is calculated using the Proportional Change in Variance (Equation 3).[4]

Equation 3: Proportional Change in Variance $\text{PCV} = \frac{\varphi_{2,B} - \varphi_{2,A}}{\varphi_{2,A}}$

Markov Chain Monte Carlo estimation

Estimates were generated using a Gibbs sampler, Rjags, from within RStudio v1.0.143. Non-informative priors, 5,000 iteration burn-in and 100,000 saved posterior samples were used. No initialisation values were used, but chains with different random starting points gave similar results, and traceplots indicated good levels of convergence and mixing. The Raftery-Lewis diagnostic indicated an appropriate number of burn-in and saved samples in order to obtain the parameters of interest with a 0.005 margin of error at the 0.025 quartile with 95% accuracy.

Point estimates are the average of the posterior samples for the parameter of interest, while uncertainty is communicated through the credible intervals (CI), the smallest interval covering 95% of posterior samples for the parameter of interest. Predicted probabilities are estimated by calculating the logged odds for each health system environment in each posterior sample using the parameters estimated in the Bayesian model described above, converting logged odds to probabilities, and averaging across posterior samples for each health system environment in order to obtain the point estimate.

References

1 Rabe-Hesketh S, Skrondal A. *Multilevel and longitudinal modelling using Stata*. Second Edi. College Station, Tex. : Stata Press 2008. doi:10.1515/9783110499469-002

2 Evans CR, Williams DR, Onnela J-P, *et al.* A multilevel approach to modeling health inequalities at the intersection of multiple social identities. *Soc Sci Med* 2017;:0–1. doi:10.1016/j.socscimed.2017.11.011

3 Merlo J. Multilevel analysis of individual heterogeneity and discriminatory accuracy (MAIHDA) within an intersectional framework. *Soc Sci Med* Published Online First: 2017. doi:10.1016/j.socscimed.2017.02.040

4 Axelsson Fisk S, Mulinari S, Wemrell M, *et al.* Chronic Obstructive Pulmonary Disease in Sweden: An intersectional multilevel analysis of individual heterogeneity and discriminatory accuracy. *SSM - Popul Heal* 2018;**4**:334–46. doi:10.1016/j.ssmph.2018.03.005

Sochas L. *BMJ Global Health* 2020; S:e002139. doi: 10.1136/bmjgh-2019-002139
